# Discovery of Potential Anti-Microbial Molecules and Spectrum Correlation Effect of *Ardisia crenata* Sims via High-Performance Liquid Chromatography Fingerprints and Molecular Docking

**DOI:** 10.3390/molecules29051178

**Published:** 2024-03-06

**Authors:** Chunli Zhao, Changbin Wang, Yongqiang Zhou, Tao Hu, Yan Zhang, Xiang Lv, Jiaxin Li, Ying Zhou

**Affiliations:** 1 College of Pharmacy, Guizhou University of Traditional Chinese Medicine, Guiyang 550025, China; zhaochunli008@gzy.edu.cn (C.Z.); ht52213267071024@126.com (T.H.); zhangyan003@gzy.edu.cn (Y.Z.); 05lvxiang@163.com (X.L.); lijiaxin048@gzy.edu.cn (J.L.); yingzhou71@126.com (Y.Z.); 2 Kunming Institute of Botany, Chinese Academy of Sciences, Kunming 650201, China; wangchangbin@mail.kib.ac.cn

**Keywords:** *Ardisia crenata* Sims, HPLC fingerprint, anti-microbial activity, spectrum-effect relationship, molecular docking

## Abstract

*Ardisia crenata* Sims, an important ethnic medicine, is recorded in the *Chinese Pharmacopoeia* for treating laryngeal diseases and upper respiratory tract infections. This study aimed to evaluate the antimicrobial effect of extracts and potential antimicrobial compounds of *A. crenata* Sims. It was found that the roots of *A. crenata* Sims have a potential inhibitory effect on *Candida albicans* and *Aspergillus flavus*, with MICs of 1.56 mg/mL and 0.39 mg/mL, and the leaves of *A. crenata* Sims have a potential inhibitory effect on *Pseudomonas aeruginosa* and *Staphylococcus aureus*, with MICs of 3.12 mg/mL and 6.77 mg/mL, respectively. Meanwhile, five compounds including one catechin and four bergenins were obtained from roots. These components were identified on the fingerprint spectrum, representing chromatographic peaks 16, 21, 22, 23, and 25, respectively. Among these, 11-*β*-d-glucopyranosyl-bergenin and (−)-gallocatechin showed potential inhibition for *Staphylococcus aureus* and *Pseudomonas aeruginosa* with MIC of 0.26 and 0.33 mg/mL, respectively. The roots, stems, and leaves of *A. crenata* Sims are very similar in chemical composition, with large differences in content. Principal component analysis (PCA) and Hierarchical cluster analysis (HCA) showed that 16 batches of *A*. *crenata* Sims could be divided into four main production areas: Guizhou, Jiangsu, Guangxi, and Jiangxi. Furthermore, molecular docking results showed that 11-*β*-d-glucopyranosyl-bergenin had a better affinity for Casein lytic proteinase P (ClpP), and (−)-gallocatechin possessed a strong affinity for LasA hydrolysis protease and LasB elastase. These findings suggest catechin and bergenins from *A. crenata* Sims can be used as antimicrobial activity molecules.

## 1. Introduction

As the most common human bacterial pathogen, *Staphylococcus aureus* often asymptotically colonizes the nasal mucosa of humans, causing superficial infections of the skin and mucosa, and even life-threatening systemic infections [1]. People with damaged skin and mucosal barrier or impaired immune systems are particularly vulnerable to *S. aureus* infection, which can cause a variety of diseases, including pneumonia, sepsis, serious skin infection, and respiratory system infection [2,3]. Among the various virulence factors of *S. aureus*, Casein Hydrolase (ClpP) is the key virulence factor that determines the pathogenicity of *S. aureus* and plays a crucial role in the pathogenicity of bacteria. Therefore, ClpP has been identified as a new candidate antibacterial target for screening and discovering inhibitors of important virulence factors of *S. aureus* [4,5]. *Pseudomonas aeruginosa* can colonize various surfaces and tissues with strong adaptability, invasiveness, and pathogenicity and cause various acute and chronic infections such as burn wounds, urinary tract infections (UTI), and lung infections [6]. This can be attributed not only to its highly endogenous nature and acquired resistance but also to various virulence factors [7]. *P. aeruginosa* can secrete different kinds of extracellular proteases, such as LasA protease, LasB elastase, alkaline protease, and protease IV. Among them, AprA and LasB can alter the mucosal cilia clearance rate, degrade lung tissue, and disrupt the host immune system, thereby strongly promoting lung diseases [8]. *P. aeruginosa* is also the main cause of chronic lung infection in patients with cystic fibrosis. The emergence and spread of widely resistant or multidrug-resistant *P. aeruginosa* isolates pose significant risks to human health [9].

Antibiotics are one of the greatest inventions of the 20th century and are widely used in the treatment of infectious diseases. However, approximately 50% of antibiotics are abused and misused globally every year, leading to strong antibiotic resistance in bacteria [10,11]. Traditional Chinese medicine (TCM) has been used clinically for thousands of years, with characteristics such as less toxicity, fewer side effects, and multiple targets of action. It has played a crucial role in human efforts to overcome major epidemics. The effective active ingredients of Chinese herbal medicine mainly contain polysaccharides, essential oils, and phenolic compounds [12,13,14]. These components often have certain antibacterial effects and are not prone to developing compound resistance, and they even reverse the compound resistance of bacteria. They have received increasing attention in clinical and scientific research [15]. *Ardisia crenata* Sims, a plant of the genus *Ardisia*, also called zhu sha gen is mainly distributed in Guizhou, China [16]. It is also used as an important Miao medicine called ba zhua jin long with antimicrobial, anti-viral, anti-inflammatory, and anti-tumor effects [17,18]. In addition, *A*. *crenata* Sims has a significant antibacterial effect on type A, type B *hemolytic streptococcus,* and *Staphylococcus aureus* [19]. Clinically, as the main drug of Kaihoujian spray (child type), it is mainly used to treat respiratory tract infections, tonsillitis, rheumatic bone pain, and other diseases without general toxicity or adverse effects [20,21]. At present, studies have found that *A. crenata* Sims mainly contains coumarins, triterpenoid saponins, flavonoids, and other chemical components [22,23]. Especially, bergenin as one kind of main coumarin, has inhibitory effects on the growth of microbes [24].

Modern analytical techniques have played an important role in the quality identification of TCM, including the detection of hydrazine in real water and soil samples from the growing areas of TCM [25]. In particular, integrated metagenomics and meta-transcriptomics sequencing can also be used to examine the abundance of microbial consortiums and their metabolites [26,27,28]. Macromolecular phase separation was also used to deliver bioactive compounds [29]. The chromatography-mass spectrometry technology can detect the active components of TCM and food, as well as analyze and confirm the structure of unknown active substances [30]. In addition, research has shown that the anti-mold secondary amine bond of soy protein can effectively and environmentally improve the anti-mold properties of its adhesive [31].

In this paper, we established the fingerprints of *A. crenata* Sims roots, stems, and leaves, and assigned their common peaks and characteristic peaks. Meanwhile, five compounds were isolated and purified from *A. crenata* Sims. The contents of these compounds were also determined to illustrate the differences in different medicinal parts of *A. crenata* Sims. Furthermore, we also compare the antimicrobial activity of different batches of *A. crenata* Sims. Moreover, these compounds were evaluated by molecular docking analysis, obtaining good antimicrobial activity to elucidate the possible mechanism.

## 2. Results and Discussion

### 2.1. Anti-Microbial Activity Evaluation of A. crenata Sims

The different parts of *A. crenata* Sims were used to evaluate antimicrobial activities against two kinds of fungi and six kinds of bacteria. As shown in Table 1, the extracts of root, stem, and leaf exhibited inhibitory activity on these test strains with an inhibition zone diameter (IZD) of 6.08~20.84 mm. The results showed that the roots of *A. crenata* Sims had good activity against *Candida albicans* and *Aspergillus flavus*. Leaves had good antibacterial activity against *Pseudomonas aeruginosa* and *Staphylococcus aureus*. In addition, the results indicated that the Minimum inhibitory concentration (MIC) values of root to *Candida albicans* and *Aspergillus flavus* were 1.56 mg/mL and 0.39 mg/mL, respectively. The MIC values of leaves against *Pseudomonas aeruginosa* and *Staphylococcus aureus* were 3.12 mg/mL and 6.77 mg/mL, respectively (Table 2).

### 2.2. Structural Identification of Known Compounds from A. crenata Sims

The known compounds (**1**–**5**) (Table 3) were identified on the basis of detailed spectroscopic interpretation (Figure S6–S8) and comparison with the previously reported data on (−)-gallocatechin (1) [32], 11-*α*-d-galactopyranoside bergenin (2) [33], 11-*β*-d-glucopyranosyl bergenin (3) [34], bergenin (4) [35] and 11-*O*-galloybergenin (5) [36]. Compounds (2), (3), and (5) were found in *A. crenata* Sims for the first time.

### 2.3. Analysis of HPLC Fingerprint

**Figure 1:**
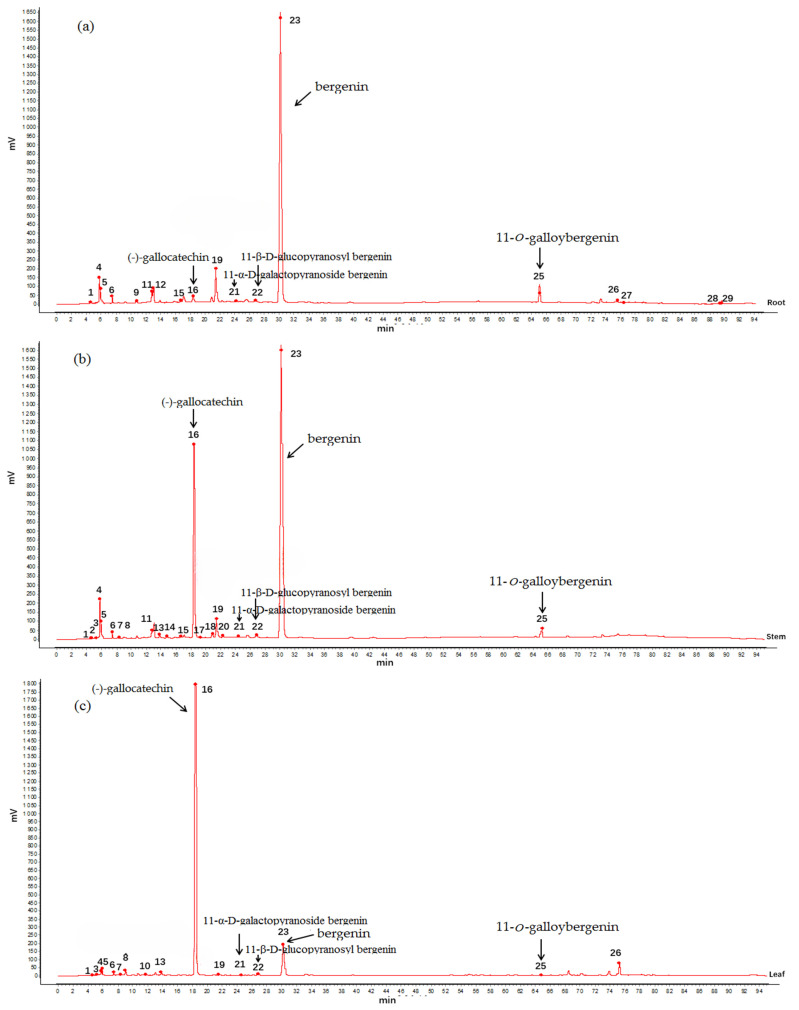
The HPLC reference fingerprint of the roots (**a**), stems (**b**), and leaves (**c**) from *A. crenata* Sims.

**Figure 2:**
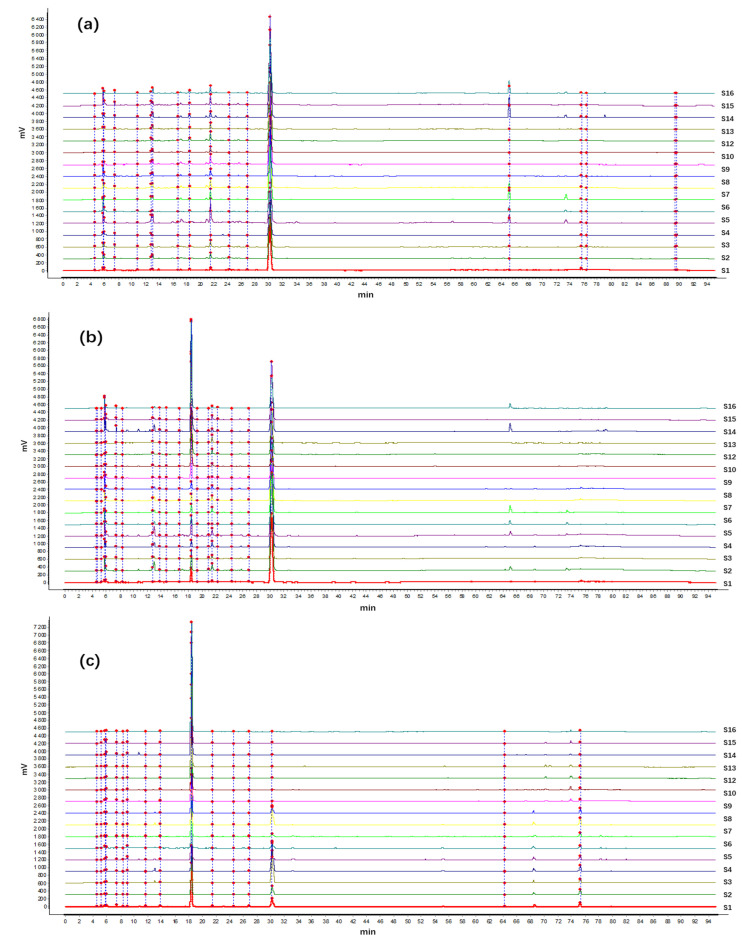
The HPLC fingerprint of the roots (**a**), stems (**b**), and leaves (**c**) from *A. crenata* Sims.

The HPLC fingerprint of 16 batches and the reference fingerprint from *A. crenata* Sims are presented in Figure 1 and Figure 2. Ten common peaks are shown as peaks 1, 4, 5, 6, 16, 19, 21, 22, 23, and 25 in Figure 1a–c. Among these, the identified compounds belonged to peaks 16, 21, 22, 23, and 25, respectively.

The similarity analysis was conducted with *A. crenata* Sims in the S1 production area as a reference (Table 4). As a result, the root similarity was more than 0.97 in different producing areas, while, many differences in the fingerprints of the leaves and stems of *A. crenata* Sims from different regions. The aboveground part of *A. crenata* Sims was different due to being affected by the environment.

### 2.4. PCA and HCA Analysis of 16 Batches of A. crenata Sims

**Figure 3:**
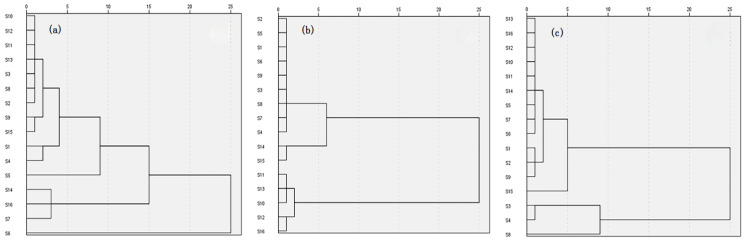
HCA analysis of roots (**a**), stems (**b**), and leaves (**c**) from *A. crenata* Sims.

**Figure 4:**
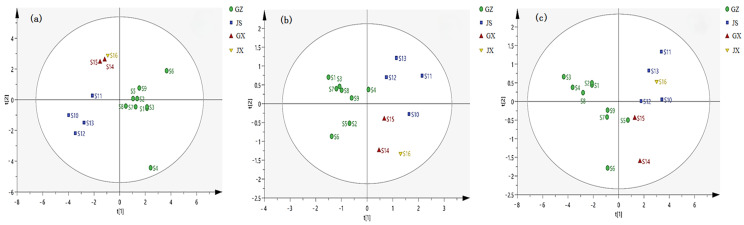
PCA analysis of roots (**a**), stems (**b**), and leaves (**c**) from *A. crenata* Sims.

As shown in Figure 3, HCA analysis found that the roots of *A. crenata* Sims from 16 production areas have high similarity, and the differences in chemical composition of stems and leaves due to different climatic environments in different production areas. The results of PCA analysis showed that the roots, stems, and leaves of *A. crenata* Sims in the four production areas could be divided into four categories, representing the major production areas, including Guizhou, Jiangsu, Guangxi, and Jiangxi (Figure 4).

### 2.5. Effect of Compounds ***1***–***5*** on Anti-Microbial Activities

The isolated and identified compounds were used to evaluate the antimicrobial activities against the tested strains. As shown in Table 5, compounds **1**–**5** exhibited inhibitory activity on these microorganisms with inhibition zone diameters (IZD) of 6.11~9.28 mm. The results showed that 11-*β*-d-glucopyranosyl-bergenin, 11-*α*-d-galactopyrnside-bergenin, and 11-*O*-galloybergenin had good activity against *Staphylococcus aureus*. (−)-gallocatechin and bergenin had good antibacterial activity against *Pseudomonas aeruginosa*. In addition, the results indicated that the MIC values of these compounds against *Staphylococcus aureus* and *Pseudomonas aeruginosa* ranged from 0.26 to 0.39 mg/mL, respectively.

### 2.6. Spectrum–Effect Relationship

The spectrum–effect relationship between chromatographic peaks and anti-microbial activity was established by GRA and PLSR models. As a result, GRA analysis showed that the correlation data of all the common peaks were greater than 0.7. This indicated that the antimicrobial activities of *A. crenata* Sims were caused by the compounds represented by all these peaks (Table 6).

**Figure 5:**
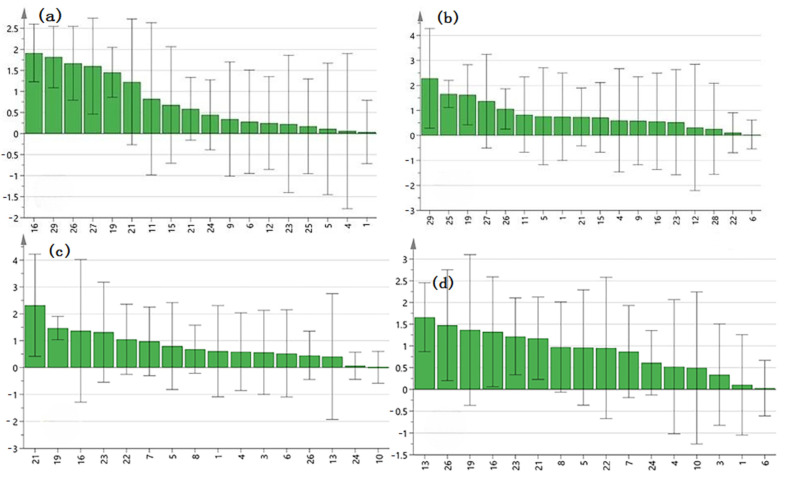
The common peaks of *A*. *crenata* Sims and the VIP values. (**a**) *Candida albicans*, (**b**) *Aspergillus flavus*, (**c**) *Pseudomonas aeruginosa*, and (**d**) *Staphylococcus aureus*.

**Figure 6:**
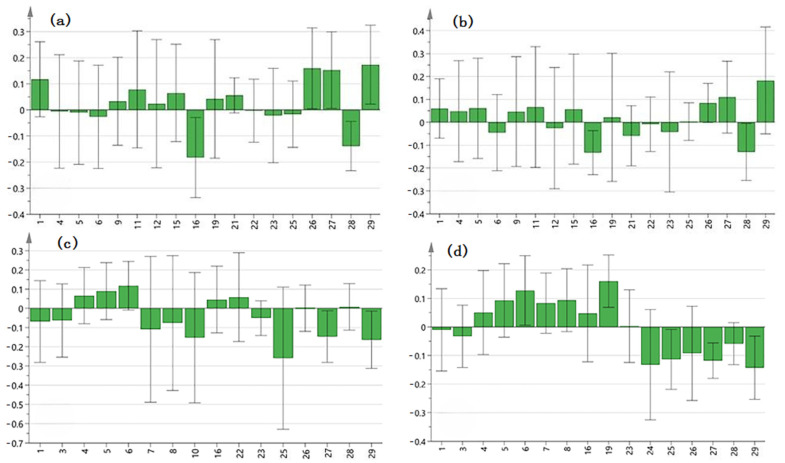
The partial expression coefficient of the common peaks. (**a**) *Candida albicans*, (**b**) *Aspergillus flavus*, (**c**) *Pseudomonas aeruginosa*, and (**d**) *Staphylococcus aureus*.

VIP value > 1 was used as the standard to screen the key components of the anti-microbial effect of roots from *A. crenata* Sims. As a result, PLSR analysis showed that peaks of 19, 21, 26, 27, and 29 were the main components of *A. crenata* Sims against *Candida albicans* (Figure 5a and Figure 6a). Peaks of 19, 25, 26, 27, and 29 were the main components of roots from *A. crenata* Sims against *Aspergillus flavus* (Figure 5b and Figure 6b). Peaks 16 and 22 were the main components of leaves from *A. crenata* Sims against *Pseudomonas aeruginosa* (Figure 5c and Figure 6c). Peaks 19, 23, and 8 were the main components of leaves from *A. crenata* Sims against *Staphylococcus aureus* (Figure 5d and Figure 6d). These results indicated that the antimicrobial effect of *A. crenata* Sims was jointly influenced by multiple components.

### 2.7. Quantitative Analysis of Anti-Microbial Ingredients in A. crenata Sims

As shown in Table 7, the HPLC analysis showed that the contents of bergenin, and (−)-gallocatechin were much higher than 5.5 mg/g in the roots. Moreover, these two components in roots were much higher quantities than in the stems and leaves. In addition, 3.84 mg/g of 11-*O*-galloybergenin was found in the leaves. Meanwhile, 0.38 mg/g of 11-*β-*d-glucopyranosyl-bergenin was found in the stems, which was much higher than the content in the roots and leaves. The chromatograms of standards solution of these compounds were showed in Figures S1–S5 (Supplementary Materials).

### 2.8. Molecular Docking of Active Compounds on Key Target Proteins of Bacteria

**Figure 7:**
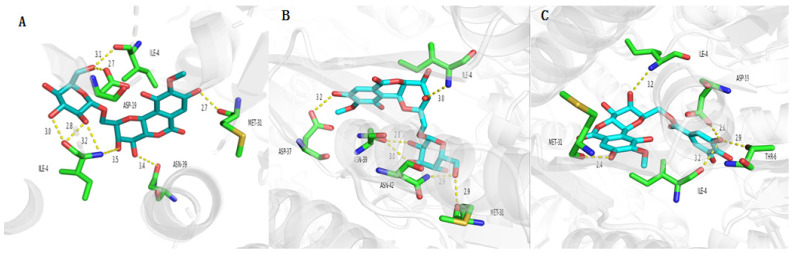
The molecular docking of 11-*β*-d-glucopyranosyl-bergenin (**A**), 11-*α*-d-galactopyranoside-bergenin (**B**), and 11-*O*-galloybergenin (**C**) on ClpP.

The ClpP protease in *S. aureus* is responsible for stress tolerance and participates in virulence regulation [37]. It plays an important role in maintaining homeostasis and pathogenicity in bacteria. Therefore, inhibition of this protease can reduce the overall virulence level of pathogenic bacteria and kill them [38]. The interaction between the active compounds and target proteins, the ClpP PRs (PDB ID: 3V5e), LasA PRs (PDB ID: 3IT7), LasB PRs (PDB ID: 3DBK), DNA ligase (2XCQ), DNA gyrase (3JSN), and MurF ligase (4CVL), with the receptors and active compounds as ligands, was explored using Schrödinger Suite. As shown in Table 8 and Figure 7, 11-*β*-d-glucopyranosyl-bergenin (A), 11-*α*-d-galactopyranoside-bergenin (B), and 11-*O*-galloybergenin (C) provided a strong binding affinity of −9.84~−8.34 to ClpP protease. Among them, eight hydrogen bonds were formed between 11-*β*-d-glucopyranosyl-bergenin and ClpP PR (Figure 7A), including between 8-hydroxyl and residue MET-31; between 4-hydroxyl and residue ASN-39; between 2′-hydroxyl, 3′-hydroxyl group of glucose, 3-hydroxyl and residue ILE-4; and between 6′-hydroxyl group of glucose and residue ILE-4, ASP-19. For 11-*α*-d-galactopyranoside-bergenin, six hydrogen bonds were formed between hydroxyl groups and residues ILE-4, ASP-37, ASN-39, ASN-42, and MET-31 (Figure 7B). For 11-*O*-galloybergenin, five hydrogen bonds were formed between hydroxyl groups and residues ILE-4, ASP-19, THR-6, and MET-31 (Figure 7C).

**Figure 8:**
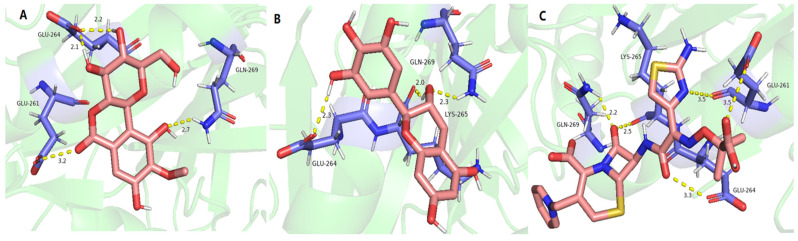
The molecular docking of bergenin (**A**), (−)-gallocatechin (**B**), and ceftazidime (**C**) on DNA gyrase.

As shown in Table 8 and Figure 8, bergenin (A), (−)-gallocatechin (B), and ceftazidime (C) provided a good binding affinity of −4.17–−5.63 to DNA gyrase. Among them, four hydrogen bonds were formed between bergenin and DNA gyrase (Figure 8A), including between 3-hydroxyl, 4-hydroxyl, and residue GLU264; between 6-carbonyl and residue GLU261; and between 10-hydroxyl and residue GLN-269. For (−)-gallocatechin, three hydrogen bonds were formed between 3-hydroxyl and residues GLN-269 and LYS-265; and between 3′-hydroxyl and residues GLU264 (Figure 8B). For the positive drug ceftazidime, five hydrogen bonds were formed between hydroxyl groups and residues GLN-269, LYS-265, GLU-261, and GLU-264 (Figure 8C).

**Figure 9:**
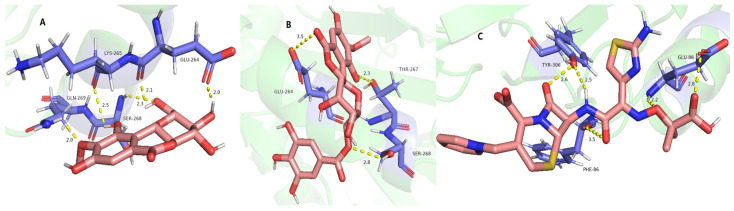
The molecular docking of bergenin (**A**), 11-*O*-galloybergenin (**B**), and ceftazidime (**C**) on DNA ligase.

As shown in Table 8 and Figure 9, bergenin (A), 11-*O*-galloybergenin (B), and ceftazidime (C) provided a good binding affinity of −4.22–−6.72 to DNA ligase. Among them, five hydrogen bonds were formed between bergenin and DNA ligase (Figure 9A), including between 3-hydroxyl and residue GLU-264; between 12-methoxy and residue GLN-269; between 10-hydroxyl and residue LYS-265; and between 11-hydroxyl and residue LYS-265, SER-268. For 11-*O*-galloybergenin, three hydrogen bonds were formed between 11-ester and residues SER-268; between 6-carbonyl and residues GLU264; and between 10-hydroxyl and residue THR-267 (Figure 9B). For the positive drug ceftazidime, five hydrogen bonds were formed between hydroxyl groups and residues THR-306, PHE-86, and GLU-88 (Figure 9C).

**Figure 10:**
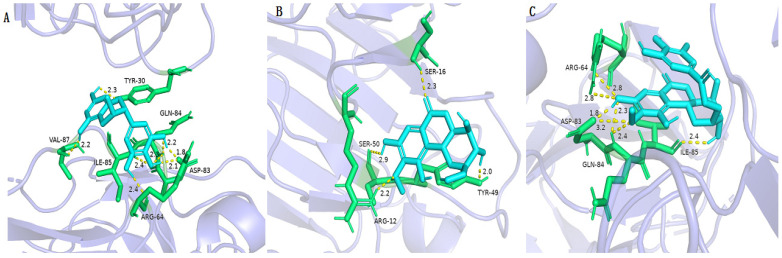
The molecular docking results of (−)-gallocatechin (**A**), bergenin (**B**), and 11-*O*-galloybergenin (**C**) on LasA.

The protease of *P. aeruginosa* is mainly regulated by the Las pathway of Quorum sensing (QS). Among them, the LasA hydrolytic protease induces respiratory infections by assisting in the colonization and digestion of host tissues [39]. For LasA PRs, as shown in Table 8 and Figure 10, (−)-gallocatechin, bergenin, and 11-*O*-galloybergenin exhibited good binding activity to LasA PRs with a docking score of more than −5.0. Among them, eight hydrogen bonds are formed between (−)-gallocatechin and LasA PRs (Figure 10A), including between 5-hydroxyl and residue VAL-87; between 7-hydroxyl and residue TYR-30; between 4′-hydroxyl and residue ILE-85, ASP-83, and GLN-84; between 3′-hydroxyl and residue GLN-84 and ASP-83; and between 5′-hydroxyl and residue ARG-64. For bergenin, four hydrogen bonds were formed between 3-hydroxyl and residue TYR-49; between 6-carbonyl and residue SER-16; between 8-hydroxyl and residue SER-50; and between 12-methoxy and residue ARG-12 (Figure 10B). For 11-*O*-galloybergenin, seven hydrogen bonds were formed between 3-hydroxyl and residue ILE-85; between 12-methoxy and residue ASP-83, GLN-84; and between 8-hydroxyl and residue ASP-83, GLN-84, and ARG-64 (Figure 10C).

**Figure 11:**
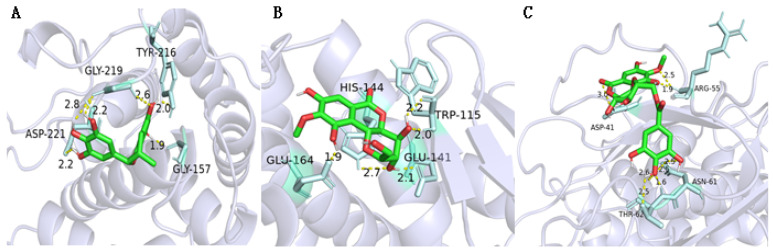
The molecular docking results of (−)-gallocatechin (**A**), bergenin (**B**), and 11-*O*-galloybergenin (**C**) on LasB.

The QS system of *P. aeruginosa* regulates the production of virulence factor LasB elastase. Additionally, it can damage and decompose human cellular tissues [8]. For LasB PRs, as shown in Table 8 and Figure 11, (−)-gallocatechin, bergenin, and 11-*O*-galloybergenin showed good binding affinity to LasB PRs with a docking score of more than −5.5. Among them, six hydrogen bonds are formed between (−)-gallocatechin and LasB PRs (Figure 11A), including between 7-hydroxyl and residue GLY-157; between 5-hydroxyl and residue TYR-216, GLY-29; between 3′, 4′-hydroxyls and residue GLY-219; and between 5′-hydroxyl and residue ASP-221. For bergenin, five hydrogen bonds were formed between 10-hydroxyl and residue GLU-164; between 4-hydroxyl and residue TRP-115; and between 3-hydroxyl and residue HIS-144, GLU-141 (Figure 11B). For 11-*O*-galloybergenin, eight hydrogen bonds were formed between 3′, 4′-hydroxyls and residue ASN-61; between 4′-hydroxyls and residue THR-62; between 6-carbonyl and residue ASP-41; and between 12-methoxy, 10-hydroxyl, and residue ARG-55 (Figure 11C).

**Figure 12:**
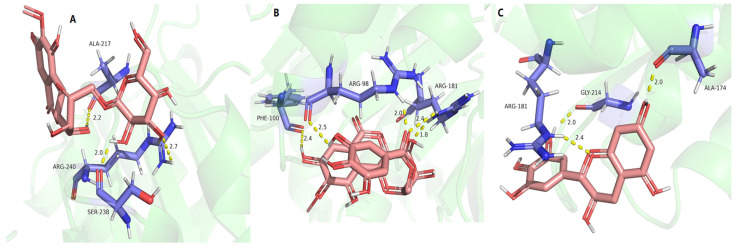
The molecular docking results of 11-*α*-d-galactopyranoside-bergenin (**A**), 11-*O*-galloybergenin (**B**), and (−)-gallocatechin (**C**) on MurF ligase.

As shown in Table 8 and Figure 12, 11-*α*-d-galactopyranoside-bergenin (A), 11-*O*-galloybergenin (B), and (−)-gallocatechin (C) provided a good binding affinity of −4.19~−5.03 to MurF ligase. Among them, three hydrogen bonds were formed between 11-*α*-d-galactopyranoside-bergenin and MurF ligase, including between 4-hydroxyl and residue ALA-217; between 2′-hydroxyl of galactose and residue ARG-240; and between 3′-hydroxyl of galactose and residue SER-238 (Figure 12A). For 11-*O*-galloybergenin, five hydrogen bonds were formed between 8-hydroxyl and residue PHE-100; between 4-hydroxyl and residue ARG-181; between 5′-hydroxyl and residue ARG-98; and between 6-carbonyl and residue ARG-98, ARG-181 (Figure 12B). For (−)-gallocatechin, three hydrogen bonds were formed between 1-oxygen and residues ARG-181; between 7-hydroxyl and residues ALA-174; and between 3′-hydroxyl and residue GLY-214 (Figure 12C).

### 2.9. Molecular Docking of Active Compounds on Key Target Proteins of Fungi

**Figure 13:**
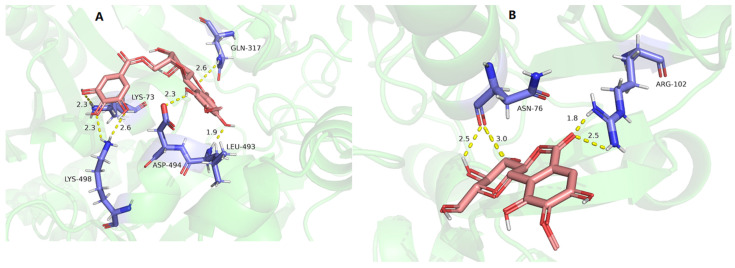
The molecular docking results of 11-*O*-galloybergenin (**A**) and bergenin (**B**) on SQS PRs.

The mechanisms of antifungal drugs mainly include inhibiting fungal cell wall synthesis and affecting cell membrane function. 1,3-β-glucan synthase and chitin synthase are the key fungal target proteins involved in the cell wall and squalene synthase (SQS) in the cell membrane. For SQS PRs, as shown in Table 9 and Figure 13, 11-*O*-galloybergenin (A), and bergenin (B) provided a good binding activity to SQS PRs with docking scores of 4.23 and 4.61, respectively. Among them, six hydrogen bonds were formed between 11-*O*-galloybergenin and SQS PRs, including between 3′, 4′-hydroxyls and residue LYS-498; between 2′-hydroxyl and residue LYS-73; between 10-hydroxyl and residue ASP-494; between 8-hydroxyl and residue LEU-493; and between 1-oxygen and residue GLN-317 (Figure 13A). For bergenin, four hydrogen bonds were formed between 3, 4-hydroxyls and residue ASN-76; and between 6-carbonyl and residue ARG-102 (Figure 13B).

**Figure 14:**
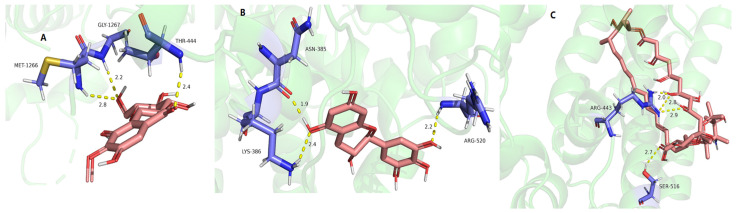
The molecular docking results of bergenin (**A**), (−)-gallocatechin (**B**) and Nystatin (**C**) on 1, 3-β-glucan synthase.

As shown in Table 9 and Figure 14, bergenin (A), (−)-gallocatechin (B), and Nystatin (C) provided a good binding affinity of −4.62–−6.38 to 1,3-β-glucan synthase. Among them, three hydrogen bonds were formed between bergenin and 1, 3-β-glucan synthase, including between 11-hydroxyl and residue MET-1266, GLY-1267; and between 6-carbonyl and residue THR-444 (Figure 14A). For (−)-gallocatechin, three hydrogen bonds were formed between 5-hydroxyl and residue LYS-384, ASN-385; and between 3′-hydroxyl and residue ARG-520 (Figure 14B). For the positive drug Nystatin, four hydrogen bonds were formed between hydroxyl groups and residues ARG-443 and SER-516 (Figure 14C).

**Figure 15:**
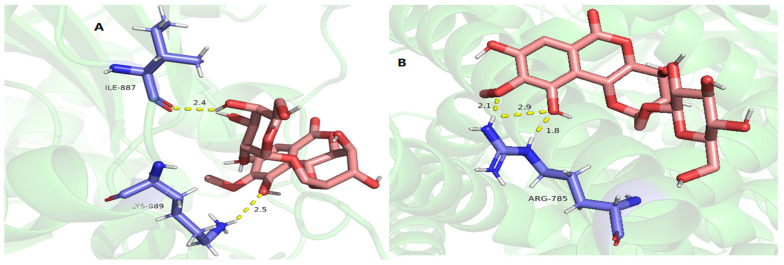
The molecular docking results of 11-*α*-d-galactopyranoside-bergenin (**A**) and 11-*β*-d-glucopyranosyl-bergenin (**B**) on chitin synthase.

As shown in Table 9 and Figure 15, 11-*α*-d-galactopyranoside-bergenin (A) and 11-*β*-d-glucopyranosyl-bergenin (B) showed high-level binding affinities of −4.13 and −4.51 for chitin synthase, respectively. Among them, two hydrogen bonds were formed between 11-*α*-d-galactopyranoside-bergenin and chitin synthase, including between 10-hydroxyl and residue LYS-889; and between 4′-hydroxyl of galactose and residue ILE-887 (Figure 15A). For 11-*β*-d-glucopyranosyl-bergenin, three hydrogen bonds were formed between 10-hydroxyl, 9-methoxy, and residue ARG-785 (Figure 15B).

## 3. Materials and Methods

### 3.1. Instruments and Chemicals

In this study, we used the following instruments: Agilent-1260 High-Performance Liquid Chromatographer (Agilent, Santa Clara, CA, USA); DRX-500 AVANCE III-600MHz superconducting nuclear magnetic resonance imager (Bruker, Bremen, Germany); RID-20A differential refractive detector (Shimadzu, Kyoto, Japan); SB-600DTY ultrasonic Multi-frequency cleaning machine (Ningbo, China); Hve-50 autoclave; HCB-1300V medical ultra clean table (Haier, Qingdao, China). We used the following chemicals and materials: 11-*α*-d-galactopyranoside-bergenin, bergenin, (−)-gallocatechin, 11-*O*-galloybergenin, and 11-*β*-d-glucopyranosyl-bergenin (standard laboratory- and self-made products); UN1648 Acetonitrile (GR, Thermo Scientific, Waltham, MA, USA); Methanol (GR, BCR, USA); Phosphoric acid (GR, Tianjin Kemel, Tianjin, China); C-18 reversed-phase column packing ODS-A-HG (YMC, Kyoto, Japan); Sephadex LH-20 (Beijing Solarbio, Beijing, China); Semi-preparative column (250 mm × 10 mm, 5 μm, Shimadzu, Kyoto, Japan): Blank drug-sensitive paper (Jining Best Micro, Jining, China); Ceftazidime (Hangzhou Microbial Reagent, Hangzhou, China); Nystatin (Hefei BASF, Hefei, China).

### 3.2. Plant Materials and Test Strains

Sixteen batches of samples from different regions of China were identified as *Ardisia crenata* Sims by Professor Shenghua Wei of Guizhou University of Traditional Chinese Medicine, as shown in Table S1. *Candida albicans* (BNCC 186382), *Aspergillus flavus* (A1142B), *Staphylococcus aureus* (ATCC 6538P), *Bacillus subtilis* (ATCC 6633), *Enterococcus faecalis* (ATCC 19433), *Escherichia coli* (CICC 10389), *Pseudomonas aeruginosa* (ATCC 9027), and *Proteus vulgaris* (ACCC 11002) were provided by the laboratory of the College of Life Sciences, Guizhou University (Guiyang, Guizhou).

### 3.3. Extraction and Isolation of Roots from A. crenata Sims

The dried roots of *A. crenata* Sims were crushed to obtain 10 kg powder, and extracted with a 70% (*v*/*v*) ethanol/water mixture at reflux three times for 2 h each. The extraction solution was extracted with petroleum ether, ethyl acetate, and n-butanol successively, and dried to obtain the petroleum ether layer (30.2 g), ethyl acetate layer (102.42 g), and n-butanol layer (879.6 g) extracts. The soluble fraction of the n-butanol (312.01 g) was eluted by dichloromethane/methanol (10:1–0:1) via silica gel column chromatography to obtain seven fractions (Fr.1~Fr.7). Compound **4** (5.6 g) was precipitated colorless crystals from Fr.1. to Fr.3 (8.27 g) was subjected to ODS column chromatography with MeOH-H_2_O (2:8 to 1:0) to obtain sub-fractions A1–A11. A2 was purified by semi-preparative HPLC (MeOH-H_2_O, 20:80; flow rate: 3 mL/min) to obtain compound **2** (19 mg, t_R_ = 8 min) and compound **3** (17 mg, t_R_ = 11 min). The soluble fraction of the ethyl acetate (87.01 g) was eluted by dichloromethane-methanol (100:1–0:1) via silica gel column chromatography to obtain 11 fractions (Fr.1~Fr.11). Fr.5 (7.24 g) was chromatographed by ODS column with MeOH-H_2_O (2:8 to 1:0) to obtain sub-fractions E1–E11. E2 was purified by semi-preparative HPLC (MeOH-H_2_O, 40:60; flow rate: 3 mL/min) to obtain compound **5** (30 mg, t_R_ = 15 min). E1 was purified by semi-preparative HPLC (MeOH-H_2_O, 20:80; flow rate: 3 mL/min) to obtain compound **1** (19 mg, t_R_ = 13 min).

### 3.4. Sample Preparation

The roots, stems, and leaves of *A. crenata* Sims were dried and crushed into powder. Sample powders (2.0 g) were accurately weighed and extracted with 50 mL methanol in a stoppered Erlenmeyer flask with an ultrasonic multi-frequency cleaning machine (frequency 40 kHz) for 40 min. The obtained extracts were filtered, concentrated under reduced pressure and vacuum dried. Then, the extracts were mixed with 10% DMSO to configure with a concentration of 100 mg/mL as the test solution for the anti-bacterial experiment. The 11-*α*-d-galactopyranoside-bergenin, 11-*β*-d-glucopyranosyl-bergenin, bergenin, 11-*O*-galloybergenin, and (−)-gallocatechin were dissolved in 30% MeOH-H_2_O to for content determination.

### 3.5. Chromatographic Conditions

Chromatography was performed on an Agilent-1260 HPLC (Agilent, USA) equipment with a diode array detector (DAD) and a YMC-Pack ODS-A (250 mm × 4. 6 mm, 5 μm) at 25 °C. The mobile phase consisted of acetonitrile (A) and 0.1% phosphoric acid (B) with a gradient elution mode as follows: 0–15 min, 5%–10% A; 15–35 min, 10%–10% A; 35–65 min, 10%–24% A; 65–80 min, 24%–40% A; 80–85 min, 40%–50% A; 85–90 min, 50%–5% A; 90–95 min, 5%–5% A. The flow rate was 0.5 mL/min with an injection volume of 5 μL and the DAD detection wavelength was set at 214 nm.

### 3.6. Validation of Methodology

The precision test was evaluated by six consecutive injections of the same sample (S3) solution, and the repeatability was evaluated by repeating six times with samples (S3) from the same place of origin. The stability tests were analyzed within 0, 2, 4, 6, 8, 10, 12, and 24 h, respectively.

### 3.7. Analysis of HPLC Fingerprint

The similarity of the Chinese medicine chromatographic fingerprints was analyzed and evaluated using the 2012A version system under the optimized HPLC conditions. Hierarchical cluster analysis (HCA) and multivariate principal component analysis (PCA) were used to divide the samples into different groups based on the similarity of their measured properties [40].

### 3.8. Anti-Bacterial Activity Evaluation

The antimicrobial doses were set according to the pharmacological dosage of *A. crenata* Sims in Kaihoujian spray (child type) and the antimicrobial concentration gradient selected in the pre-experiment. The blank drug-sensitive paper (6 mm × 1 mm) was soaked in the compounds solution (0.4 mg/mL) and extracts solution of roots, stems, and leaves (100 mg/mL) of *A. crenata* Sims and the sterile 10% DMSO solution was used as the blank control. The ceftazidime and nystatin were set as positive drugs with a concentration of 1.0 mg/mL. *Candida albicans* and *Aspergillus flavus* were placed in an incubator at 28 °C while the other six bacteria were cultured in an incubator at 37 °C for 24 h to observe the growth of the bacteria and fungi. The diameter of the inhibition zone was measured three times, and the data were recorded.

The minimum inhibitory concentration (MIC) of extracts and compounds from *A. crenata* Sims was performed according to the two-fold serial dilution method. The dilution concentrations of roots, stems and leaves extracts for *S. aureus* were 55.50 to 0.22, 66.90 to 0.26, and 54.20 to 0.21 mg/mL, respectively. The dilution concentrations of roots, stems and leaves extracts for *B. subtilis* were 55.00 to 0.21, 66.30 to 0.26, and 60.20 to 0.24 mg/mL, respectively. The dilution concentrations of root, stem, and leaf extracts for *E. faecalis* were 66.60 to 0.26, 71.40 to 0.28, and 70.60 to 0.27 mg/mL, respectively. The dilution concentrations of root, stem, and leaf extracts for *E. coli* were 66.60 to 0.26, 71.40 to 0.28, and 70.60 to 0.27 mg/mL, respectively. The dilution concentrations of root, stem, and leaf extracts for *P. aeruginosa* were 54.70 to 0.21, 60.40 to 0.24, and 100.00 to 0.19 mg/mL, respectively. The dilution concentrations of root, stem, and leaf extracts for *P. vulgaris* were 67.80 to 0.26, 67.50 to 0.26, and 55.50 to 0.22 mg/mL, respectively. The dilution concentrations of root, stem, and leaf extracts for *C. albicans* were 100 to 0.19, 98 to 0.19, and 100 to 0.19 mg/mL, respectively. The dilution concentrations of root, stem, and leaf extracts for *A. flavus* were 100 to 0.19, 88.6 to 0.34, and 98.8 to 0.38 mg/mL, respectively. The dilution concentration of 11-*β*-d-glucopyranosyl-bergenin for *S. aureus* was 1.04 to 0.03 mg/mL and (−)-gallocatechin for *P. aeruginosa* was 1.32 to 0.04 mg/mL. The dilution concentration of ceftazidime for bacteria was 0.64 to 0.02 mg/mL and nystatin for fungi was 0.84 to 0.025 mg/mL. Bacteria were cultured at 37 °C for 24 h and fungi were kept at 28 °C for 48 h, respectively. Then, 10 μL of 2,3,5-triphenyl tetrazolium chloride (TTC) was added to the plates and incubated. The MIC was determined as the highest dilution of extracts and compounds exhibiting no growth visibility of bacteria and fungi. All the tests were performed in replicates three times.

### 3.9. Spectrum-Effect Relationship

#### 3.9.1. Gray Relational Analysis (GRA)

GRA analysis could be used to determine the contribution of fingerprint-shared peaks to anti-microbial activity. Sixteen batches of *A. crenata* Sims were used as reference sequences to determine the inhibition zone diameter of the four sensitive strains. The common peak area data in the HPLC fingerprint of the corresponding batches of *A. crenata* Sims were taken as the comparison sequence. The gray correlation analysis method was used to establish a spectral efficacy correlation mathematical statistical model and the correlation degree of each common peak to the efficacy indicators was calculated, and the resolution coefficient was ξ = 0.5 [41].

#### 3.9.2. Partial Least Squares Regression (PLSR)

The peak area of each common peak in the fingerprint of *A. crenata* Sims was set as the independent variable (X) and the antibacterial activity of *A. crenata* Sims against strains as the dependent variable (Y), using these, the regression models were built sequentially. Then SIMCA-P 14 was used for PLSR analysis and the regression coefficient of X to Y and the variable importance projection (VIP) value were calculated [42].

### 3.10. Molecular Docking Analysis

Molecular docking is currently one of the important means for studying the interaction between small molecules and proteins in traditional Chinese medicine. It can be used to identify targets with a high affinity for speculating the mechanism of traditional Chinese medicine in treating diseases. In this paper, the key target proteins related to antimicrobial activity were used for docking with ingredients of *A. crenata* Sims. The molecular docking analyses of anti-microbial compounds to target proteins were conducted according to the Ligand docking module of Schrödinger Suite 2021-1 (Schrödinger, LLC, New York, NY, USA). The Crystal structures of ClpP PR (PDB ID: 3V5e), LasA PR (PDB ID: 3IT7), LasB PR (PDB ID: 3DBK), DNA ligase (2XCQ), DNA gyrase (3JSN) and MurF ligase (4CVL) were chosen for the docking analysis [43].

## 4. Conclusions

In summary, we innovatively established fingerprints, for the first time, of the roots, stems, and leaves of *A. crenata* Sims from different origins, and screened the main regions as sources of medicinal materials. In addition, we also speculated on the antimicrobial active ingredients in the chemical composition of *A. crenata* Sims via combining antimicrobial experiments with fingerprint analysis. Furthermore, we isolated and identified five phenolic compounds and quantified them from *A. crenata* Sims. Among these, 11-*β*-d-glucopyranosyl-bergenin and (−)-gallocatechin showed better inhibition for *Staphylococcus aureus* and *Pseudomonas aeruginosa*, respectively. Moreover, 11-*β*-d-glucopyranosyl-bergenin had a much better affinity to ClpP PR and (−)-gallocatechin showed the best affinity to LasA PR and LasB PR. These results confirmed that unvalidated molecular docking analysis may suggest a possible mechanism of antimicrobial activity. And the phenolic components could be used as anti-microbial activity molecules for developing new anti-microbial agents. In our next work, we will conduct molecular biology experiments to verify the antimicrobial target proteins screened by molecular docking.

## Figures and Tables

**Table 1:** Anti-microbial activities of different parts from *A. crenata* Sims (x ± s, *n* = 3, d, mm).

No.	Strains	Root (100 mg/mL)	Stem (100 mg/mL)	Leaf (100 mg/mL)	Ceftazidime ^a^ (1.0 mg/mL)
1	*Staphylococcus aureus* (ATCC 6538P)	8.08 ± 0.27	6.50 ± 0.33	8.98 ± 1.56	22.89 ± 2.78
2	*Bacillus subtilis* (ATCC 6633)	8.60 ± 1.19	6.54 ± 0.23	7.13 ± 0.72	30.07 ± 3.11
3	*Enterococcus faecalis* (ATCC 19433)	6.15 ± 0.08	6.04 ± 0.02	6.10 ± 0.07	26.42 ± 2.71
4	*Escherichia coli* (CICC 10389)	6.10 ± 0.06	6.41 ± 0.12	6.89 ± 0.68	29.57 ± 2.63
5	*Pseudomonas aeruginosa* (ATCC 9027)	8.83 ± 0.11	7.13 ± 0.89	10.48 ± 1.97	28.00 ± 2.74
6	*Proteus vulgaris* (ACCC 11002)	6.42 ± 0.27	6.46 ± 0.32	8.71 ± 1.35	23.85 ± 2.89
					**Nystatin ^b^** (1.0 mg/mL)
7	*Candida albicans* (BNCC 186382)	10.89 ± 1.58	6.11 ± 0.03	6.08 ± 0.54	25.75 ± 2.67
8	*Aspergillus flavus* (A1142B)	20.84 ± 1.76	7.25 ± 0.62	6.15 ± 0.08	12.27 ± 1.32

^a,b^ Positive control.

**Table 2:** MIC of different parts from *A. crenata* Sims on different strains (x ± s, *n* = 3, mg/mL).

No.	Strains	Root	Stem	Leaf	Ceftazidime ^a^
1	*Staphylococcus aureus* (ATCC 6538P)	6.94 ± 0.41	8.36 ± 0.29	6.77 ± 0.37	0.16 ± 0.01
2	*Bacillus subtilis* (ATCC 6633)	6.87 ± 0.12	8.29 ± 0.24	7.53 ± 0.33	0.04 ± 0.003
3	*Enterococcus faecalis* (ATCC 19433)	8.33 ± 0.31	8.93 ± 0.16	8.82 ± 0.18	0.08 ± 0.005
4	*Escherichia coli* (CICC 10389)	8.85 ± 0.14	8.41 ± 0.23	8.11 ± 0.12	0.04 ± 0.003
5	*Pseudomonas aeruginosa* (ATCC 9027)	6.84 ± 0.15	7.55 ± 0.34	3.12 ± 0.11	0.08 ± 0.006
6	*Proteus vulgaris* (ACCC 11002)	8.47 ± 0.24	8.44 ± 0.32	6.94 ± 0.24	0.16 ± 0.02
					**Nystatin ^b^**
7	*Candida albicans* (BNCC 186382)	1.56 ± 0.15	3.06 ± 0.24	3.13 ± 0.12	0.05 ± 0.004
8	*Aspergillus flavus* (A1142B)	0.39 ± 0.023	2.77 ± 0.015	3.09 ± 0.11	0.21 ± 0.02
8	*Aspergillus flavus* (A1142B)	20.84 ± 1.76	7.25 ± 0.62	6.15 ± 0.08	12.27 ± 1.32

^a,b^ Positive control.

**Table 3:** Structures of compounds **1**–**5** from the roots of *A. crenata* Sims.

No.	R_t_ [min]	Name	Molecular Weight	Structures	Formula
1	19.25	(−)-gallocatechin	306.27	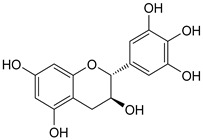	C_15_H_14_O_7_
2	23.84	11-*α*-d-galactopyranoside bergenin	490.41	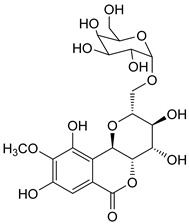	C_20_H_26_O_14_
3	27.13	11-*β*-d-glucopyranosyl bergenin	490.41	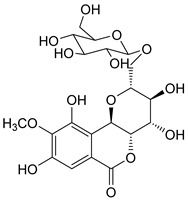	C_20_H_26_O_14_
4	30.57	bergenin	328.27	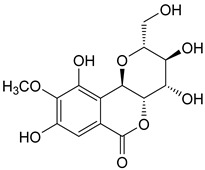	C_14_H_16_O_9_
5	62.72	11-*O*-galloybergenin	480.38	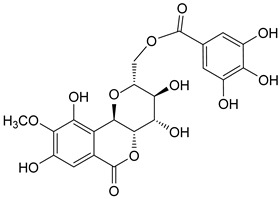	C_21_H_20_O_13_

**Table 4:** The fingerprint similarities of root, stem, and leaf from *A. crenata* Sims.

Batch	Root	Stem	Leaf
S1	1.000	1.000	1.000
S2	0.998	0.997	0.997
S3	0.999	0.994	0.580
S4	0.998	0.620	0.674
S5	0.988	0.997	0.975
S6	0.979	0.996	0.986
S7	0.983	0.996	0.979
S8	0.999	0.995	0.920
S9	0.998	0.996	0.995
S10	0.998	0.543	0.959
S11	0.998	0.543	0.959
S12	0.998	0.694	0.751
S13	0.999	0.528	0.959
S14	0.987	0.890	0.700
S15	0.997	0.904	0.600
S16	0.992	0.643	0.775
Reference fingerprint	0.998	0.972	0.980

**Table 5:** Anti-microbial activities of compounds from *A. crenata* Sims (x ± s, *n* = 3, d, mm).

Microorganisms	11-*β*-d-Glucopyranosyl-bergenin	11-*α*-d-Galacto pyrnside-bergenin	11-*O*-Galloybergenin	Bergenin	(−)-Gallocatechin	Ceftazidime ^a^
*Staphylococcus aureus* (ATCC 6538P)	9.28 ± 0.93	8.13 ± 0.88	7.91 ± 0.91	6.83 ± 0.74	6.54 ± 0.66	22.73 ± 2.66
*Bacillus subtilis* (ATCC 6633)	-	-	-	-	6.28 ± 0.68	30.02 ± 3.32
*Enterococcus faecalis* (ATCC 19433)	-	-	-	6.22 ± 0.75	-	26.31 ± 2.88
*Escherichia coli* (CICC 10389)	6.23 ± 0.72	6.08 ± 0.61	-	-	-	29.42 ± 2.77
*Pseudomonas aeruginosa* (ATCC 9027)	6.19 ± 0.74	6.41 ± 0.78	7.18 ± 0.87	6.11 ± 0.78	8.23 ± 0.89	28.13 ± 2.81
*Proteus vulgaris* (ACCC 11002)	-	-	6.11 ± 0.85	-	-	23.52 ± 2.68
						**Nystatin ^b^**
*Candida albicans* (BNCC 186382)	-	-	6.34 ± 0.76	6.74 ± 0.92	-	25.61 ± 2.46
*Aspergillus flavus* (A1142B)	-	-	6.17 ± 0.75	6.21 ± 0.83	-	12.39 ± 1.24

^a,b^ Positive control. “^–^” No activity.

**Table 6:** Grey correlation analysis between the fingerprint of *A. crenata* Sims and anti-microbial activity.

Peak Number	Root		Peak Number	Leaf	
	*Candida* *albicans*	*Aspergillus* *flavus*		*Pseudomonas aeruginosa*	*Staphylococcus* *aureus*
1	0.77	0.75	1	0.83	0.82
4	0.79	0.78	3	0.84	0.83
5	0.82	0.83	4	0.82	0.81
6	0.88	0.88	5	0.83	0.85
9	0.82	0.82	6	0.84	0.87
11	0.82	0.82	7	0.72	0.76
12	0.85	0.84	8	0.77	0.75
15	0.83	0.83	10	0.73	0.74
16	0.78	0.79	13	0.88	0.92
19	0.83	0.84	16	0.86	0.85
21	0.81	0.79	19	0.80	0.73
22	0.84	0.84	21	0.73	0.78
23	0.87	0.90	22	0.86	0.84
25	0.73	0.71	23	0.72	0.71
26	0.89	0.86	24	0.81	0.79
27	0.89	0.88	26	0.75	0.73
28	0.68	0.70			
29	0.87	0.84			

**Table 7:** The contents of active ingredients in different parts (mg/g) of *A. crenata* Sims.

No.	R_t_ (min)	Name	Root	Stem	Leaf	Regression Equation
1	19.25	(−)-gallocatechin	5.55	3.36	2.82	Y = 13,065x − 294.98 R^2^ = 0.9995
2	23.84	11-*α*-d-galactopyranoside-bergenin	0.01	-	-	Y = 8866x + 92.139 R^2^ = 0.9994
3	27.13	11-*β*-d-glucopyranosyl-bergenin	0.21	0.38	0.16	Y = 13,851x + 105.93 R^2^ = 0.9993
4	30.57	bergenin	19.11	17.80	8.10	Y = 42,625x + 310.13 R^2^ = 0.9991
5	62.72	11-*O*-galloybergenin	2.01	1.48	3.84	Y = 28,407x − 1084.7 R^2^ = 0.9990

-: not detected under the current conditions.

**Table 8:** Molecular docking score of the active compounds on key target proteins of bacteria.

Compounds	ClpP PRs (3V5e)	LasA PRs (3IT7)	LasB PRs (3DBK)	DNA Gyrase (2XCQ)	DNA Ligase (3JSN)	MurF Ligase (4CVL)
11-*β*-d-glucopyranosyl-bergenin	−9.84	−1.87	−3.58	−1.18	−1.82	−1.53
11-*α*-d-galactopyranoside-bergenin	−8.58	−2.93	−3.90	−1.56	−2.28	−4.19
11-*O*-galloybergenin	−8.34	−5.08	−5.66	−3.41	−4.91	−4.73
bergenin	−7.79	−5.49	−5.59	−4.17	−4.22	−1.21
(−)-gallocatechin	−7.65	−6.61	−6.81	−4.55	−1.14	−5.03
Ceftazidime ^a^	−3.98	−3.17	−3.86	−5.63	−6.72	−3.19

^a^ Positive control.

**Table 9:** Molecular docking score of the active compounds on key target proteins of *fungi*.

Compounds	SQS PRs (7WG1)	1,3-β-Glucan Synthase (8JZN)	Chitin Synthase (7STL)
11-*β*-d-glucopyranosyl-bergenin	−1.39	−2.23	−4.51
11-*α*-d-galactopyranoside-bergenin	−1.52	−2.42	−4.13
11-*O*-galloybergenin	−4.23	−1.18	−2.92
bergenin	−4.61	−4.93	−2.56
(−)-gallocatechin	−3.02	−4.62	−1.33
Nystatin ^a^	−3.19	−6.38	−3.11

^a^ Positive control.

## Data Availability

Data are contained within the article and Supplementary Materials.

## Supplementary Materials

The following are available online at https://www.mdpi.com/article/10.3390/molecules29051178/s1, Figure S1: The chromatogram of standards solution of bergenin; Figure S2: The chromatogram of standards solution of (−)-gallocatechin; Figure S3: The chromatogram of standards solution of 11-*α*-d-galactopyranoside-bergenin; Figure S4: The chromatogram of standards solution of 11-*β*-d-glucopyranosyl-bergenin; Figure S5: The chromatogram of standards solution of 11-*O*-galloybergenin; Figure S6: The HR-ESI-MS spectrum of 11-*α*-d-galactopyranosidebergenin; Figure S7: The HR-ESI-MS spectrum of 11-*β*-d-glucopyranosyl-bergenin; Figure S8: The HR-ESI-MS spectrum of 11-*O*-galloybergenin; Table S1: Origin of *Ardisia crenata* Sims.
