# Supplementary material for: Discovery of Potential Anti-Microbial Molecules and Spectrum Correlation Effect of Ardisia crenata Sims via High-Performance Liquid Chromatography Fingerprints and Molecular Docking
Source: Molecules. 2024 Mar 6;29(5):1178. doi: 10.3390/molecules29051178 (PMC10934372; doi:10.3390/molecules29051178)
Supplement: Supplementary file 1 [file molecules-29-01178-s001.zip › molecules-2848377-supplementary.pdf]

molecules

## Supplementary Materials

# Discovery of Potential Anti-Microbial Molecules and Spectrum Correlation Effect of *Ardisia crenata* Sims via High-Performance Liquid Chromatography Fingerprints and Molecular Docking

**Chunli Zhao [1,†], Changbin Wang [2,†], Yongqiang Zhou [1,*], Tao Hu [1], Yan Zhang [1], Xiang Lv [1], Jiaxin Li [1] and Ying Zhou [1]**

[1] College of Pharmacy, Guizhou University of Traditional Chinese Medicine, Guiyang 550025, China
[2] Kunming Institute of Botany, Chinese Academy of Sciences, Kunming 650201, China
* Correspondence: zhouyongqiang041@gzy.edu.cn
† These authors contributed equally to this work.

Figure S1. The chromatogram of standards solution of bergenin………………………….…….3
Figure S2. The chromatogram of standards solution of (-)-gallocatechin…………………….….3
Figure S3. The chromatogram of standards solution of 11-*α*-D-galactopyranoside-bergenin..….4
Figure S4. The chromatogram of standards solution of 11-*β*-D-glucopyranosyl-bergenin............4
Figure S5. The chromatogram of standards solution of 11-*O*-galloybergenin…………………...5
Figure S6. The HR-ESI-MS spectrum of11-*α*-D-galactopyranosidebergenin……………….…6
Figure S7. The HR-ESI-MS spectrum of11-*β*-D-glucopyranosyl-bergenin…………..………7
Figure S8. The HR-ESI-MS spectrum of11-*O*-galloybergenin……………………………….…8
Table S1. Origin of *Ardisia crenata* Sims……………………………………………………..9

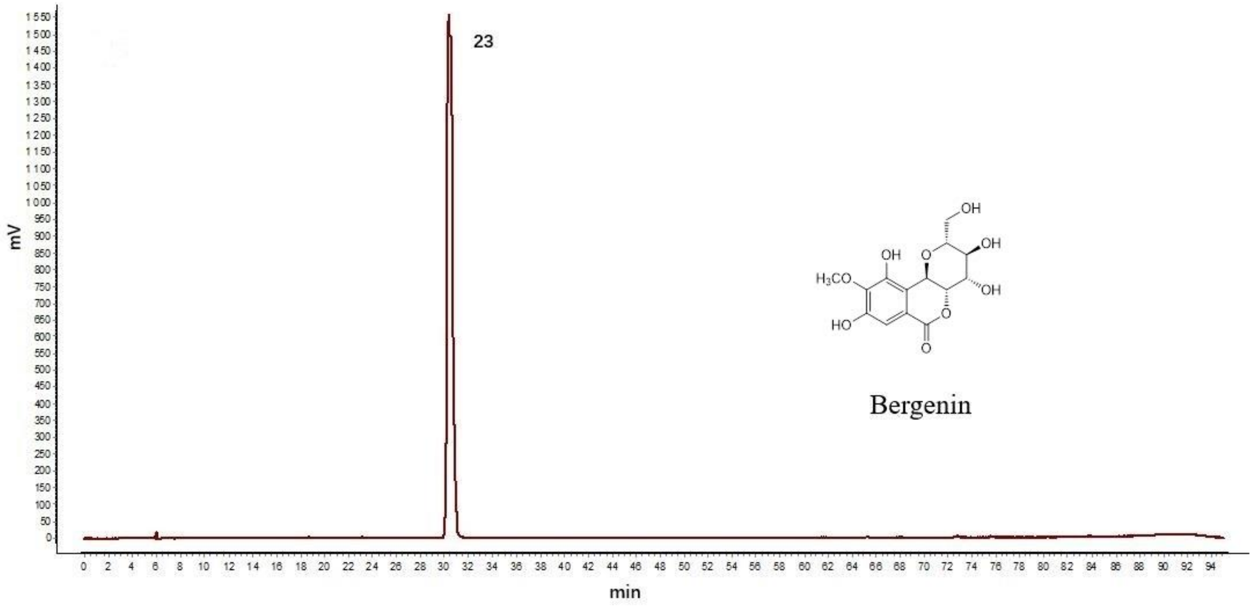


Figure S1. The chromatogram of standard solution of bergenin

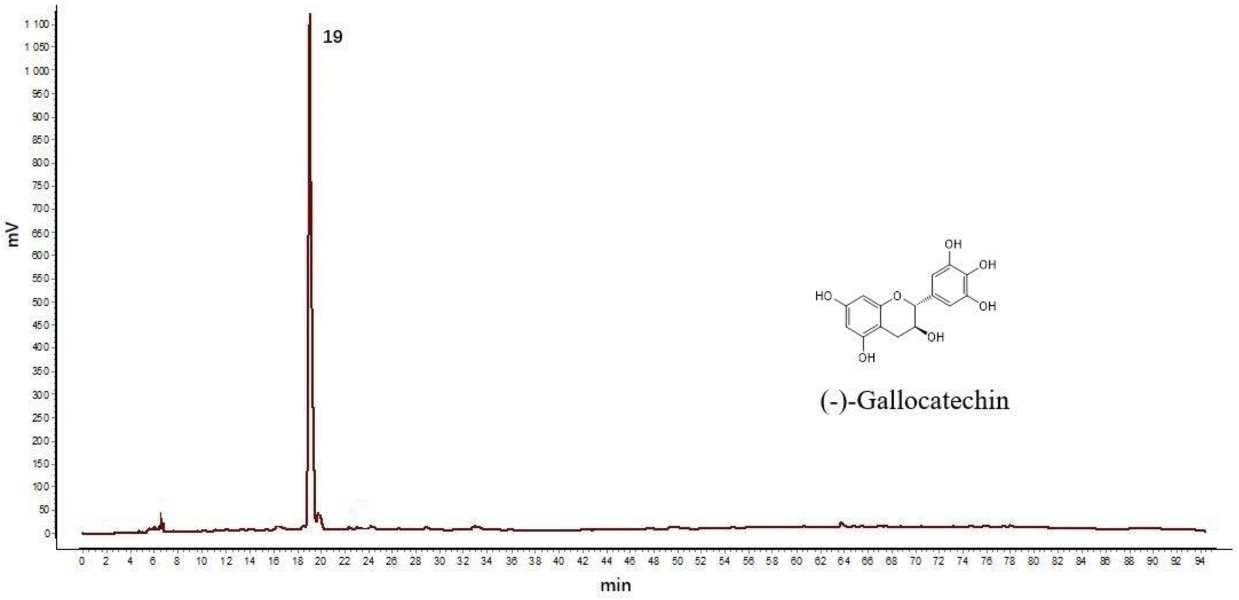


Figure S2. The chromatogram of standard solution of (-)-gallocatechin

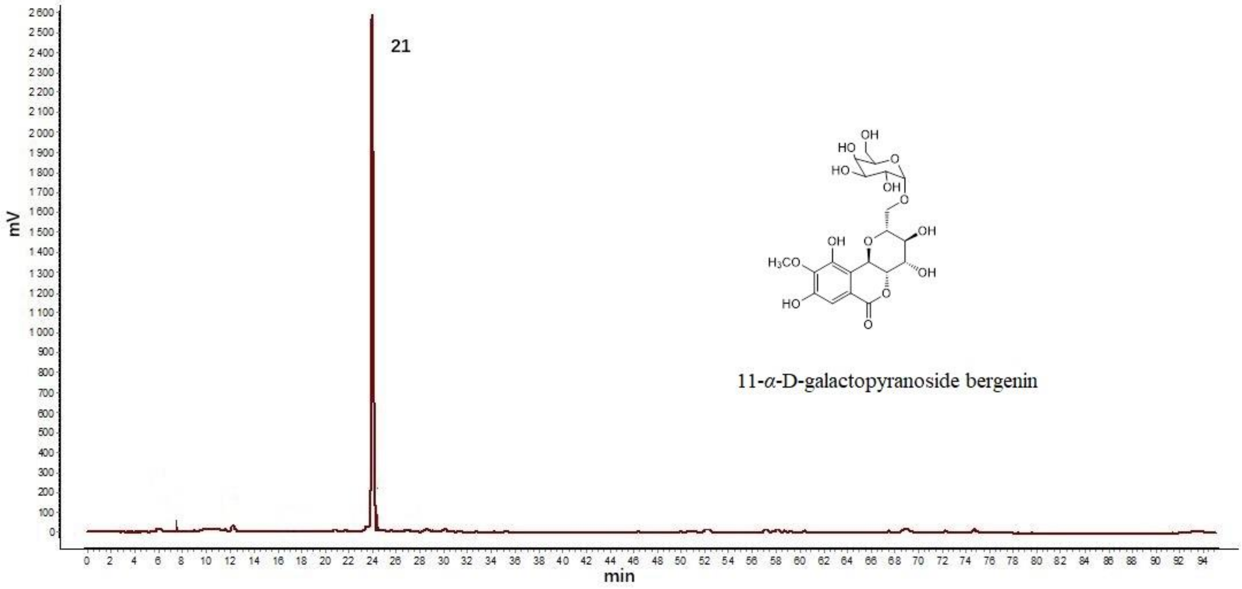


Figure S3. The chromatogram of standard solution of 11-$\alpha$-D-galactopyranoside-bergenin

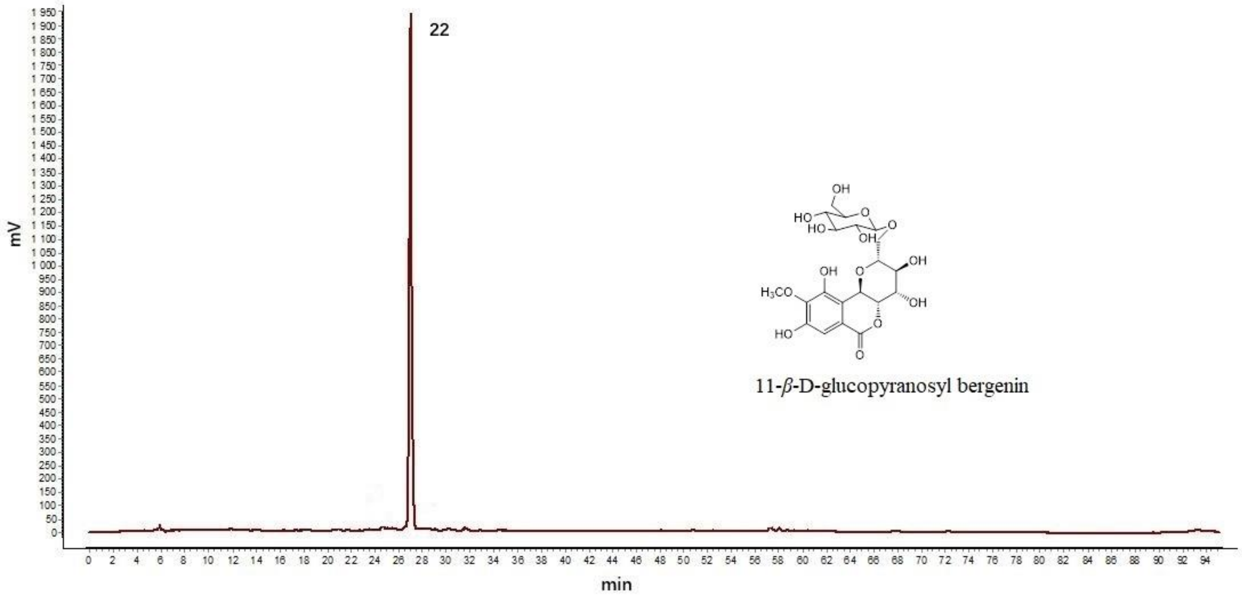


Figure S4. The chromatogram of standard solution of 11-$\beta$-D-glucopyranosyl-bergenin

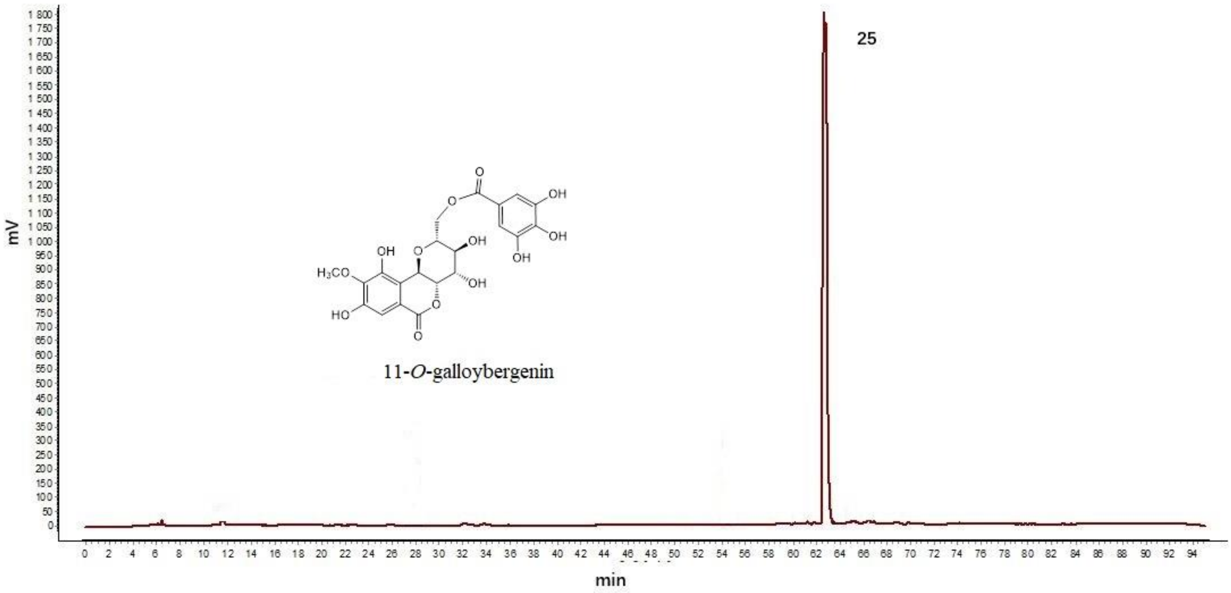


Figure S5. The chromatogram of standard solution of 11-*O*-galloybergenin

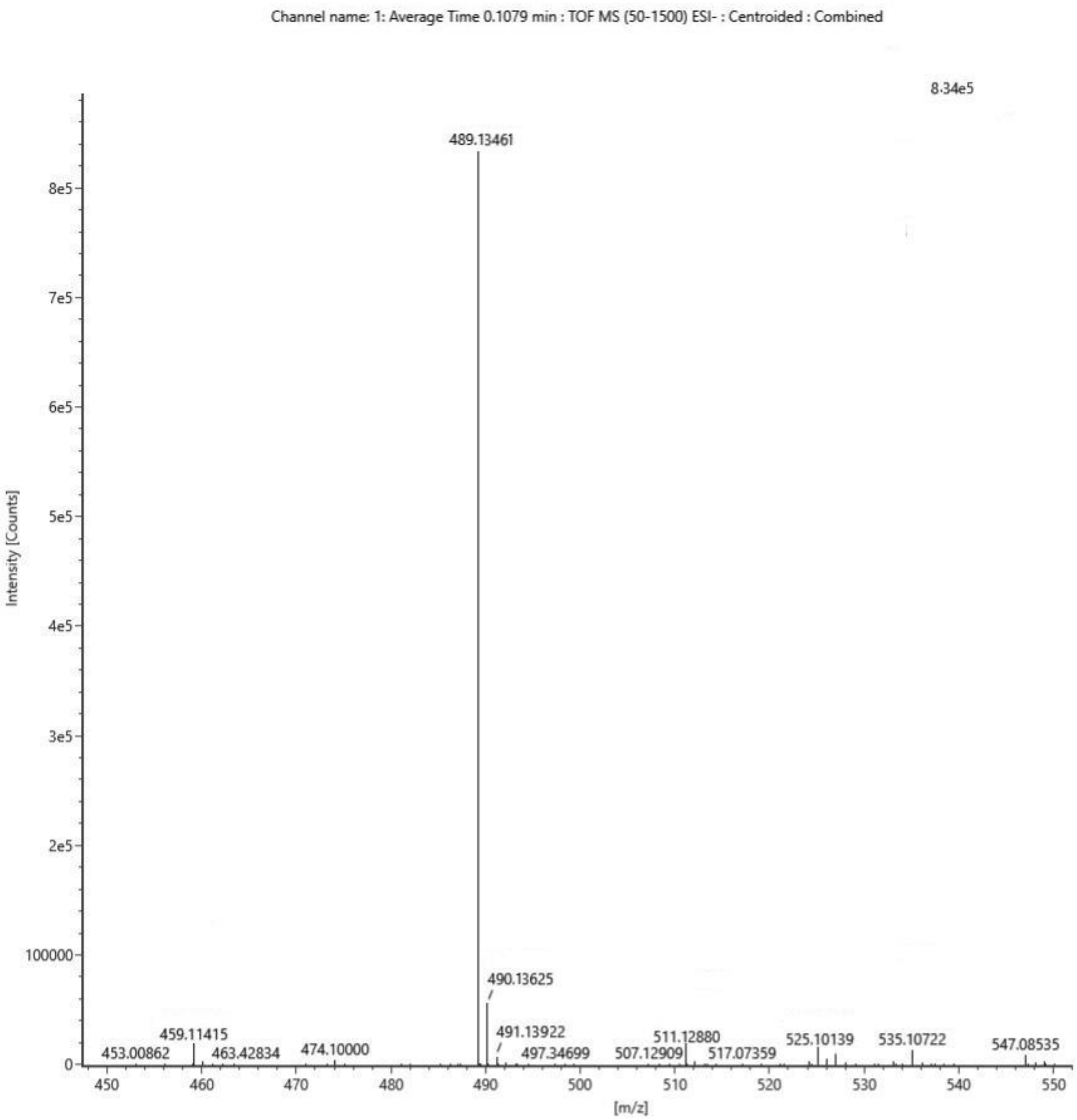


Figure S6. The HR-ESI-MS spectrum of 11-$\alpha$-D-galactopyranoside bergenin

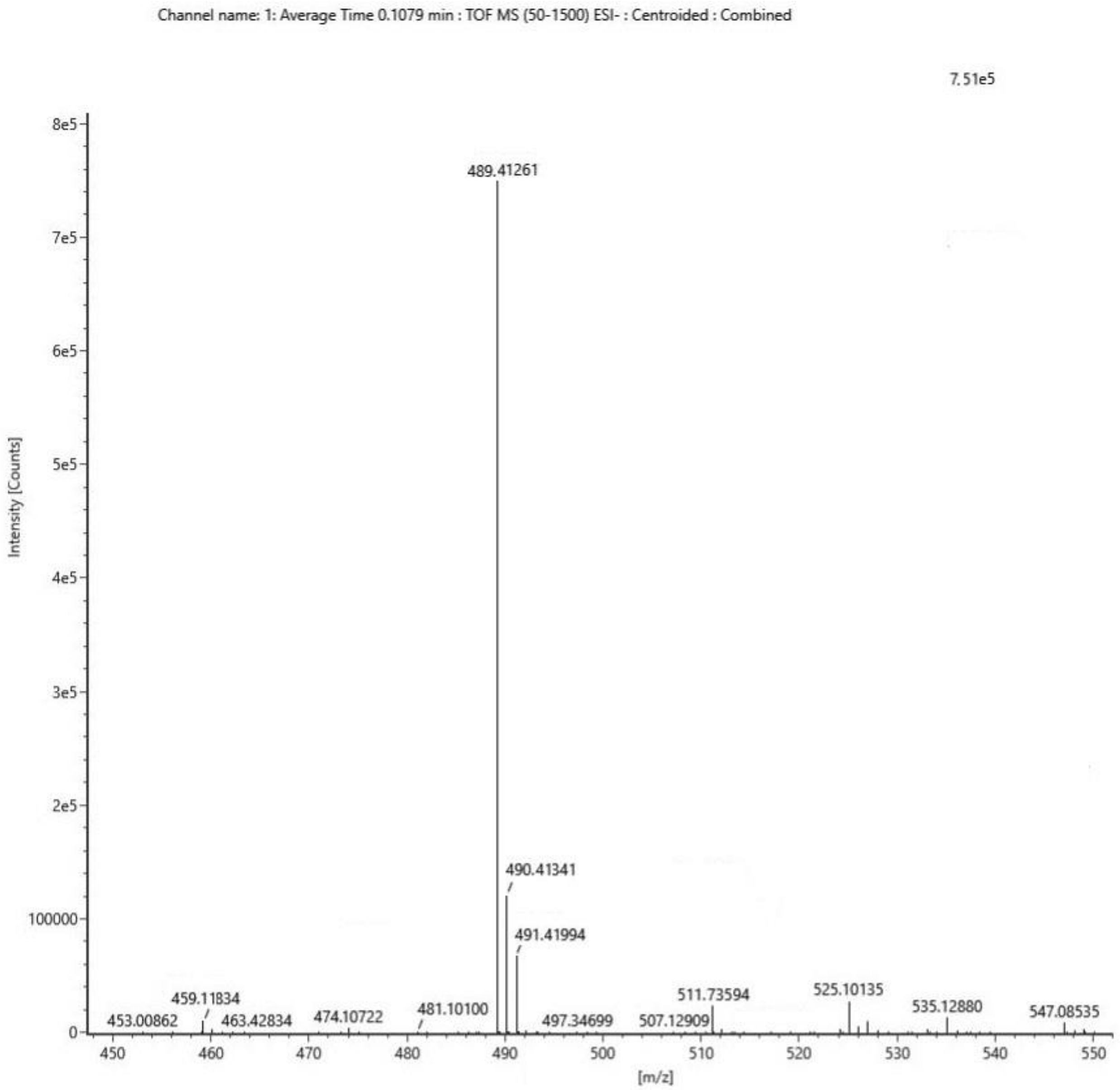


Figure S7. The HR-ESI-MS spectrum of 11-$\beta$-D-glucopyranosyl-bergenin

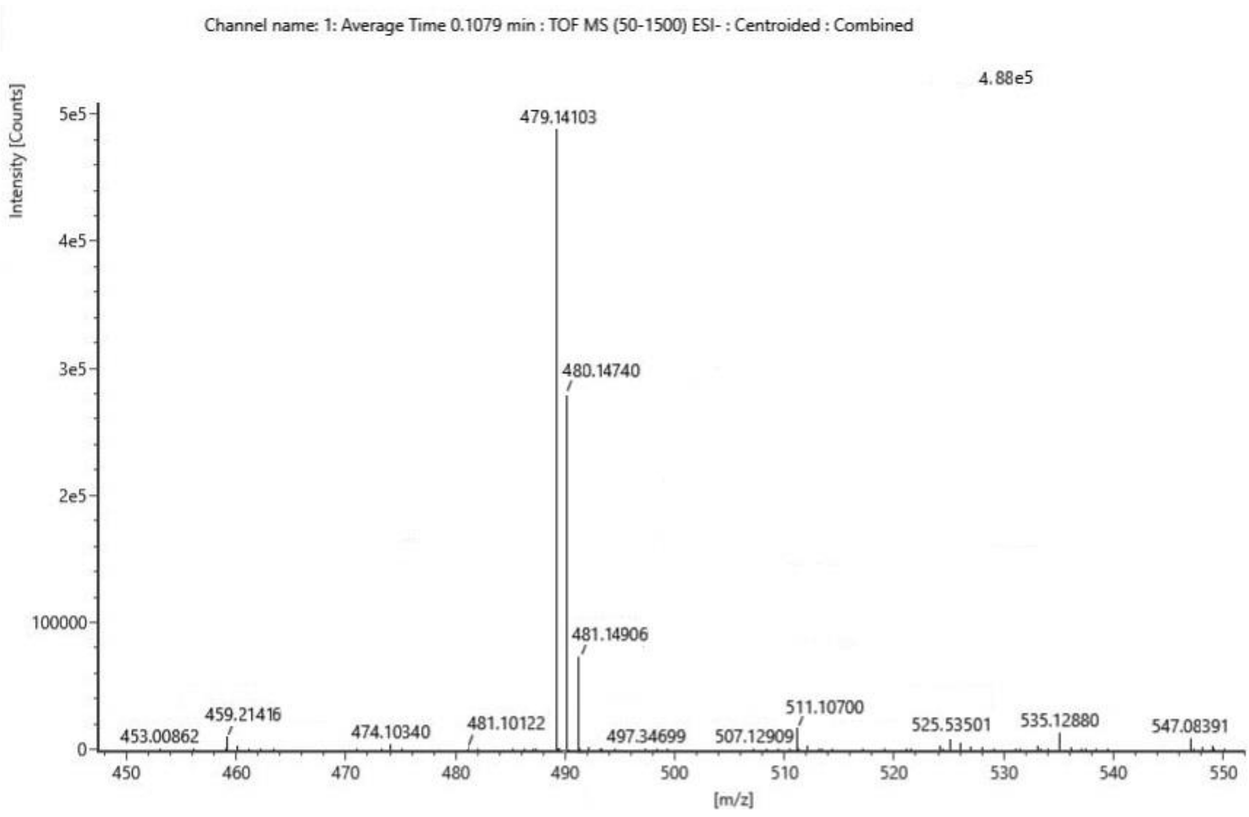


Figure S8. The HR-ESI-MS spectrum of 11-*O*-galloybergenin

Table S1. Origin of *Ardisia crenata* Sims

| Numbering | Lot number | Source of medicinal materials |
|---|---|---|
| S1 | 20190331 | Changshun County, Qiannan Prefecture, Guizhou Province |
| S2 | 20190405 | Wudang District, Guiyang City, Guizhou Province |
| S3 | 20190625 | Nanming District, Guiyang City, Guizhou Province |
| S4 | 20190723 | Kaiyang County, Guiyang City, Guizhou Province |
| S5 | 20190901 | Huaxi District, Guiyang City, Guizhou Province |
| S6 | 20190922 | Huaxi District, Guiyang City, Guizhou Province |
| S7 | 20190922 | Maling Town, Xingyi City, Guizhou Province |
| S8 | 20191005 | Nanming District, Guiyang City, Guizhou Province |
| S9 | 20191018 | Guanling County, Anshun City, Guizhou Province |
| S10 | 20190711 | Muyang County, Suqian City, Jiangsu Province |
| S11 | 20190721 | Xinhe Town, Suqian City, Jiangsu Province |
| S12 | 20190722 | Yanji Town, Suqian City, Jiangsu Province |
| S13 | 20190722 | Huaiyin District, Huaian City, Jiangsu Province |
| S14 | 20190710 | Ziliang Town, Yulin City, Guangxi Province |
| S15 | 20190723 | Rong'an County, Liuzhou City, Guangxi Province |
| S16 | 20190723 | Pingfu Township, Shangyou City, Jiangxi Province |
